# Cell assembly detection with frequent item set mining

**DOI:** 10.1186/1471-2202-13-S1-P126

**Published:** 2012-07-16

**Authors:** Christian Borgelt, David Picado, Denise Berger, George Gerstein, Sonja Grün

**Affiliations:** 1 European Centre for Soft Computing, Calle Gonzalo Gutiérrez Quirós s/n, 33600 Mieres (Asturias), Spain; 2 Laboratory of Neuromotor Physiology, IRCCS Fondazione Santa Lucia, Via Ardeatina 306, 00179 Roma, Italy; 3 Dept. of Neuroscience, 215 Stemmler Hall, University of Pennsylvania, Philadelphia PA 19104, USA; 4 Institute of Neuroscience and Medicine (INM-6), Research Center Jülich, Jülich, Germany; 5 Theoretical Systems Neurobiology, RWTH Aachen University, Aachen, Germany

## 

Hebb (1949) suggested cell assemblies as the building blocks of information processing in the brain. The member neurons are assumed to show correlated activity. Advances in multi-electrode technology providing simultaneous recordings from 100 or more neurons increased chances to identify such ensembles considerably. We present a data mining method that detects assemblies in massively parallel spike data both reliably and efficiently.

Gerstein *et al. *[1] developed an accretion approach to detect joint spiking patterns in parallel spike trains. Starting from single neurons, it iteratively accretes neurons into sequences as long as another neuron shows significantly correlated activity (χ^2^ test) with the accreted neurons (represented by their coincident spikes). However, accretion suffers from several drawbacks: it works on sequences instead of sets, thus incurring high costs from redundant detections (memory consumption, speed), and it may miss assemblies, since it needs a significantly correlated pair to start from and limits the branching factor to two sequences. We present and test an alternative approach based on frequent item set mining (FIM) that amends these drawbacks and is also conceptually neater.

FIM was originally developed for market basket analysis and aims at finding sets of products that are frequently bought together. Conceptually this is the same as finding neurons that (frequently) fire together. FIM algorithms efficiently count joint spiking events that exceed a given minimal support (occurrence frequency) by eliminating all redundancy. Found patterns may be assessed statistically by taking the maximum *p*-value over all one-neuron-against-rest tests, for which we compared χ^2^, Yates-corrected χ^2^, G-statistic and Fisher's exact test. In addition, subset conditions may be considered to reduce false positives. We examined (a) no subset conditions, (b) weak subset conditions (existence of a stepwise significant sequence as in accretion), and (c) strong subset conditions (all possible sequences must be stepwise significant). Note that (b) with χ^2^ is very similar to accretion, except that accretion executes only one test one-neuron-against-rest for the full set (which is fixed by the sequence followed). Due to accretion's redundant search this is effectively the same as taking the minimum of the *p*-values.

We generated data sets of 1s duration with 1ms resolution (i.e., 1000 time bins) and 100 independent Poisson spike trains with stationary and non-stationary rate profiles (sinusoidal or phasic-tonic, [2]). False positive (FP) tests: we counted the reported assemblies per size and number of joint spiking events (averaged over 1000 data sets). Not surprisingly, more FPs are found for higher average firing rate (37.75Hz vs. 18.875Hz) and non-stationary processes. However, they rarely exceed size 3 and 3 coincidences. FPs are lowest for strong subset requirements and depended only marginally on the used statistics. False negative (FN) tests: we injected synchronous spiking events (2-7 coincidences) of a small assembly (3-7 neurons) and counted how often this group or a superset was detected (in 1000 data sets). FNs occur mostly for 2 coincidence and 3 neurons, rarely for 3 coincidences and here only for higher firing rates and small assembly sizes. FNs are considerably more and have larger sizes and more coincidences, the stricter the subset condition. The percentage of true positive patterns with excess neurons (usually one) increases with the number of injected coincidences. Since no redundant search is carried out, FIM is much faster than accretion and does not require reducing the results to unique sets.

In summary, FIM without subset requirements, even without any statistical test, leads to essentially the same results as with subset requirements and as accretion. However, FIM uses a neater test concept (worst p-value of all one-neuron-against-rest tests) and eliminates all redundancy from the search. Introducing subset conditions reduces FPs, but increases FNs. We recommend to use FIM without subset conditions and a minimum support of 3, which reduces the number of excess neurons in the true positives. Since FIM is fast and reliable, it will allow us to apply it in a time resolved manner to detect the dynamics assembly processing [3].

## References

[B1] Gerstein GL Perkel DH Subramanian FH Identification of functionally related neural assemblies Brain Research 1978 140 1 43 62 10.1016/0006-8993(78)90237-8 203363

[B2] Louis S Borgelt C Grün S Generation and selection of surrogate methods for correlation analysis Analysis of Parallel Spike Trains 2010 Springer-Verlag, Berlin, Germany 359 382

[B3] Kilavik BE Roux S Ponce-Alvarez A Confais J Grün S Riehle A Long-term modifications in motor cortical dynamics induced by intensive practice J Neurosci 2009 29 40 12653 12663 10.1523/JNEUROSCI.1554-09.2009 19812340 PMC6665105

